# Relationship between Obesity and Dental Caries in Saudi Male Adolescents

**DOI:** 10.1155/2020/8811974

**Published:** 2020-10-08

**Authors:** Asim Al-Ansari, Muhammad Nazir

**Affiliations:** Department of Preventive Dental Sciences, College of Dentistry, Imam Abdulrahman Bin Faisal University, P.O. Box 1982, Dammam 31441, Saudi Arabia

## Abstract

**Introduction:**

Obesity and dental caries are global public health problems. There are conflicting reports about the relationship between caries and obesity. Therefore, the study aimed to investigate the association between obesity and dental caries among male adolescents.

**Materials and Methods:**

This cross-sectional study included a sample of 258 male students' aged 12 to 15 years from schools in Dammam/Al-Khobar, Saudi Arabia. The study involved measuring caries prevalence and DMFT estimates, assessing body mass index (BMI), and administering a self-completion questionnaire. Independent samples Student's *t-*test, one-way ANOVA test, Pearson's correlation test, and bivariate and multivariate logistic regression analyses were performed.

**Results:**

Caries prevalence of the sample was 79.8%, and the mean score of DMFT was 3.55 ± 2.94. The mean BMI of participants was 23.42 ± 6.82 and 18% were obese (BMI > 30). The obese participants had a higher mean DMFT score (4.46 ± 3.54) than nonobese participants (3.35 ± 2.77) (*P*=0.021). Similarly, the mean untreated caries was higher in obese (4.17 ± 3.22) than in nonobese participants (3.01 ± 2.66) (*P*=0.010). In the logistic regression analysis, after controlling for father's education, family history of obesity, meals per day, fast food per week, and physical activity in the final model, the participants with high caries experience (DMFT = 5–15) were 2.21 times more likely to have obesity than those with low caries experience (DMFT = 0–4) (*P*=0.04). No/school education of father (odds ratio 3.54, *P*=0.011), family history of obesity (odds ratio 3.27, *P*=0.002), and not performing physical activity (odds ratio 4.37, *P*=0.002) were significantly associated with an increased likelihood of obesity.

**Conclusion:**

The prevalence of caries and obesity was high in male adolescents in Saudi Arabia. Obesity was significantly associated with untreated caries and caries experience. Children with high caries experience were more likely to have obesity than children with low caries experience. Preventive programs and policies should address public health issues related to caries and obesity in male teenagers.

## 1. Introduction

Obesity is an alarming public health crisis worldwide which results from complex interaction of cultural, social, and economic factors [1]. Globally, overweight/obesity has increased dramatically from 4% in 1975 to 18% in 2016 and there were 340 million overweight/obese children and adolescents in 2016 [2]. In Saudi Arabia, a national survey estimated the prevalence of overweight (26.6%) and obesity (10.6%) in adolescents [3]. Recently, a study reported that the prevalence of obesity was 18.2% in adolescents in Riyadh in 2019 [4]. Adolescence obesity prevalence is even more profound (27.5%) in the Eastern province of the country [5]. Overweight and obese children can have several systemic disorders such as high blood pressure, impaired glucose intolerance, respiratory problems, psychological disturbance, and poor quality of life [2, 4, 6]. Nearly 77% of children persist obesity into their adulthood with an increased likelihood of disability and premature death [2, 7].

Likewise, untreated dental caries in permanent dentition affected 2.4 billion people worldwide in 2015 and its prevalence was the highest in adolescents [8]. In Saudi Arabia, the researchers estimated a caries prevalence of 70% in adolescents in a nation-wide study [9]. However, caries affected 68% of children aged 10–12 years in the Eastern province of Saudi Arabia [10]. Caries is known to cause pain and infection and can affect food intake, eating behaviors, and growth and development in children. Difficulty in chewing, speech problems, poor attendance, and impaired learning in schools can result from the complications of dental caries in children [11, 12].

There is a large body of evidence about an association between obesity and dental caries in children and adolescents [13–21]. Larsson et al. reported a positive relationship between obesity and caries in 15 years old Swedish adolescents [13]. Willershausen et al. observed a correlation between increased body weights and higher estimates of dental caries in 6 to 11 years old German children [14]. Bailleul-Forestier et al. found a significant association between body mass index (BMI) and decay, missing, and filled teeth (DMFT) index in severely obese adolescents in France [15]. In Saudi Arabia, Ashour et al. observed a strong association between caries and obesity among special care female school children in Makkah [17]. On the other hand, studies also contradict a possible relationship between dental caries and obesity [16, 18–21]. Hence, researchers stressed the need to conduct further studies to explore the link between obesity and caries [19].

A high prevalence of caries can have adverse effects on the oral and general health and well-being of adolescents. Likewise, the burden of obesity among children is alarming, and its health and social complications can be detrimental to them. The problem of obesity and its association with dental caries has been investigated in some regions of Saudi Arabia [16, 17]. However, inconsistent and mixed results on the topic emphasized the need to investigate the possible association between obesity and dental caries among adolescents. Therefore, the aim of the study was to investigate the association between obesity and dental caries among male school children in the Eastern province, Saudi Arabia.

## 2. Materials and Methods

According to the World Health Organization (WHO) guidelines, a sample of 12 years old children is more reliable, and caries prevalence studies conducted on 15 years old children can provide more meaningful data [22]. Therefore, the students (12–15 years) were recruited from middle schools in Dammam/Al-Khobar. The sample size calculation was based on assumptions: 95% level of confidence, 73% expected proportion of subjects with variable of interest (caries), and 5% precision. These calculations produced a sample of 306 children for the study. The permission to conduct this cross-sectional study was obtained from school authorities. The school children who agreed to voluntary participation and returned a signed parental consent form were eligible to participate in the study. The Committee for Biological and Medical Ethics at Imam Abdulrahman Bin Faisal University granted ethical approval of the study. Ethical standards were maintained during the conduct of the study in accordance with the Declaration of Helsinki.

Obesity among children and adolescents (2 to 19 years) can be reliably evaluated by measuring their body mass index (BMI) [23]. BMI of the children was determined by calculating their weight in kilograms and height in meters using a formula BMI = kg/m^2^. The physician's scale was used to measure the weight of the study participants while height was determined using a stadiometer [19]. Using the International Obesity Task Force cutoff values, children having BMI less than 25 kg/m^2^ were classified as normal weight, children with BMI 25–29.9 kg/m^2^ as overweight, and children with BMI equal to or greater than 30 kg/m^2^ as obese [24].

The oral examination of the participants followed WHO guidelines, and three calibrated examiners performed the diagnosis of dental caries using the disposable glove, mask, dental mirror, dental probe/explorer, gauze, tongue depressor, and an artificial light source [22]. Inter-examiner reliability for caries assessment was ensured by calculating the appropriate values of Cohen's kappa coefficient (0.78). Similarly, intra-examiner reproducibility assessed the consistency of each examiner (Cohen's kappa coefficient ranged from 0.76 to 0.81). DMFT index was used to assess decay (D), missing (M), and filled (F) teeth among the study participants. Radiographic examination was not performed. The students diagnosed with dental caries and/or dental disorders were referred for further evaluation and treatment. Likewise, obese children were advised to consult their physicians. The students also responded to a self-administered questionnaire which included questions about demographic information, obese family member, and dietary practices. Pretesting of the questionnaire was done to ensure respondents' ease of understanding the questions and to provide reliable responses.

Statistical analyses included both descriptive and inferential statistics. Descriptive statistics included frequency distributions, percentages, means, and standard deviations. Frequency distributions of overweight, obese, and healthy children were calculated and displayed in graphic form. Mean and standard deviations were calculated for untreated caries, DMFT, and BMI scores. Inferential statistical analyses included independent samples Student's *t*-tests and one-way ANOVA tests. DMFT data were categorized into low/moderate caries experience (DMFT 0–4) and high caries experience (DMFT 5–15), and data on obesity were grouped into obese (MBI > 30) and nonobese (BMI ≤ 30) to evaluate the association between obesity and caries experience [25]. Bivariate and multivariate logistic regression analyses were performed to evaluate the association between caries experience and obesity (“obese” denoted as “1” and “nonobese” denoted as “0” in the analysis). Pearson's correlation test was conducted to assess a correlation between BMI and DMFT. Statistical Package for Social Sciences (SPSS) version 22 was used to perform statistical analyses. Statistical significance involved using a *P* value of less than 0.05.

## 3. Results

There were 306 students invited to participate in the study; however, completed data of 258 students were included in the analysis and the response rate of the study was 84.3%. The mean of the participants was 14.29 ± 1.11 years. Less than half the sample (43.8%) belonged to high-income class, and 33.3% of the participants had college/university educated fathers. The family history of systemic disease (heart disease, diabetes, chronic liver disease, etc.) was reported by 39.1% of the participants. Caries prevalence of the sample was 79.8%, and the mean DMFT was 3.55 ± 2.94. The mean BMI of the sample was 23.42 ± 6.82 (Table 1).

**Figure 1:**
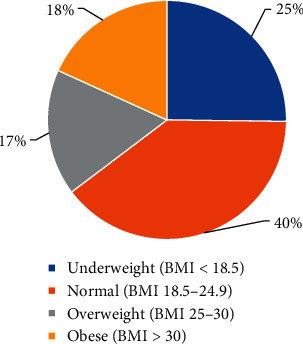
Distribution of participants according to four categories of BMI.

Based on the BMI index, 17% of participants were overweight and 18% were obese (Figure 1). Mean DMFT did not significantly differ among underweight (3.04 ± 2.77), normal weight (3.61 ± 2.8), overweight (3.27 ± 2.7), and obese (4.38 ± 3.53) (*P*=0.106). On the other hand, untreated caries significantly differed among underweight (2.71 ± 2.55), normal weight (3.31 ± 2.71), overweight (2.82 ± 2.69), and obese (4.11 ± 3.22) (*P*=0.046). The mean DMFT score differed significantly between obese (4.46 ± 3.54) and nonobese (underweight, normal weight, and overweight) participants (3.35 ± 2.77) (*P*=0.021). Similarly, the obese compared with nonobese participants had significantly higher mean untreated caries (*P*=0.010) (Table 2). The study also showed a significant positive correlation between BMI and DMFT (*r* 0.126, *P*=0.043).

In bivariate logistic analysis, no/school education of fathers (odds ratio 3.31, 95% CI 1.41, 7.75), high caries experience (odds ratio 2.34, 95% CI 1.22, 4.51), having an obese member in the family (odds ratio 2.6, 95% CI 1.35, 4.98), and not performing physical activity (odds ratio 4.32, 95% CI 1.88, 9.94) were associated with increased odds of obesity in the study participants. High caries experience was identified as a significant independent factor associated with obesity after adjusting for father's education, monthly family income, family history of obesity and systemic disease, physical activity, and consumption of food and soft drink. The participants with high caries experience were significantly 2.33 times more likely to have obesity than those with low caries experience (*P* =0.032). Furthermore, no/school education of fathers, having an obese member in the family, and not performing physical activity remained significant factors associated with an increased likelihood of having obesity (Table 3).

Table 4 shows the results of multiple logistic regression final model (backward stepwise (likelihood ratio)). After controlling for other factors in the model, high caries experience remained a significant factor associated with increased odds of obesity (odds ratio 2.21, 95% CI 1.04, 4.71). Other significant factors associated with obesity included no/school education of fathers (odds ratio 3.54, 95% CI 1.34, 9.37), having an obese member in the family (odds ratio 3.27, 95% CI 1.55, 6.89), not performing physical activity (odds ratio 4.37, 95% CI 1.73, 11.07), and having 4 and more meals per day (odds ratio 0.217, 95% CI 0.08, 0.62).

## 4. Discussion

Our hypothesis was that male children with higher BMI would experience greater dental caries. Our results confirmed this hypothesis. Dental caries and obesity were highly prevalent in our sample of male adolescents, and there was a significant association between obesity and caries experience. The findings of our study are important for the prevention and control of caries and obesity in male adolescents. In addition, an emphasis on adolescence is key to the success of public health interventions because this life span offers opportunities for behavior modifications which can lead to the establishment of health patterns into adulthood [26]. The prevalence of caries in our sample was 79.8%, which was higher than that reported in a previous study in Dammam where 68% of 10–12 years old school children had caries and male students also demonstrated high caries experience than female children [10]. High caries in our study could be related to the inclusion of male participants only, difference in study participants and sample size, and changes in caries pattern over time.

The prevalence of obesity was 18% in our study. The investigators of a study in Riyadh reported obesity in 12% of male adolescents [4]. On the contrary, a previous study on caries and obesity reported that 51% of male adolescents had obesity in Jeddah [16]. Likewise, a similar study in Madinah showed that 30% of 12-year-old-school male children were obese [27]. However, the data based on the National Health Profile Project revealed 11.2% national prevalence of obesity in male adolescents in Saudi Arabia [3]. The discrepancies in the prevalence estimates reported in these studies in the country are most likely because of differences in the measurement of obesity using different cutoff values based on the World Health Organization criteria, the Center for Disease Control (CDC) growth reference, and International Obesity Task Force [3, 4].

The findings of our study showed that students who did not perform physical activity were 4.37 times more likely to have obesity than those who performed physical activity. Similarly, low education of father (OR 3.54) and family history of obesity (OR 3.27) were also significantly associated with obesity in our participants. Moreover, the consumption of fast food 4 or more times per week was associated with increased odds of having obesity in our study, although the association was not significant. In Saudi Arabia, the economic development during the last 30 years has raised the living standards of people and created a majority of its population belonging to middle or high socioeconomic classes. As a result of financial, social, and cultural changes in the country, there occurred marked changes in the health behaviors and lifestyles of people which predisposed them to more sedentary lifestyle, lack of physical activity, more time spent on watching television and using electronic gadgets, and unhealthy dietary habits including increased energy and fat intake [4, 16].

The obese children in our study demonstrated significantly higher caries experience and untreated treated caries than nonobese children. The study also showed a significant association between obesity and caries experience. Several studies conducted in different parts of the world also established a significant relationship between overweight/obesity with increased caries experience in children and adolescents [13–15, 28]. The literature also shows studies with no significant or inverse relationship between overweight/obesity and dental caries in children in the US, [18] China, [19] Iran, [20] and Kuwait [21]. In addition, the analysis of BMI and DMFT data in children from 117 countries showed no significant correlation between BMI and caries experience at the level of countries [29]. A recent systematic review of studies conducted in Saudi Arabia indicated a positive association between BMI and caries in two studies, whereas six studies showed a negative association and a nonsignificant association in two studies on children [30]. For instance, a study of special care female children pointed out a strong association between obesity and dental caries [17]. On the other hand, Farsi et al. found no association between BMI and dental caries in the permanent dentition of children in Jeddah [16]. Peng et al. evaluated associations between caries experience and different forms of obesity (general, central, and peripheral obesity) in 12-year-old children in Australia. General obesity was measured by weight-height ratio and body mass index, central obesity by waist circumference and waist-hip ratio, and peripheral obesity by triceps skinfold thickness. The authors reported significant associations between caries experience and central and peripheral obesity; however, caries had no significant association with general obesity [31]. Therefore, the selection of surrogates of obesity may also contribute to the discrepancies in the results of studies on association between caries and obesity.

The studies on the association between BMI and caries show mixed results; however, a positive association between BMI and caries in permanent dentition is more robust and consistent than in primary dentition. This may be because both obesity and caries accumulate gradually in the life course, so a greater burden of these diseases with increasing age could result in a stronger association [32]. Our study also found a significant association between obesity and dental caries in permanent teeth.

The literature indicates that increased weight may neither contribute to a greater burden of caries nor high caries may predispose to overweight/obesity, rather common risk factors possibly account for increased likelihood of an association between BMI and caries [17]. There are modifiable and nonmodifiable risk factors common to obesity and caries such as biological, genetic, dietary, lifestyle behavior, and environmental and socioeconomic factors [17, 30, and 33]. Biological and development factors (developmental conditions), sociodemographic and household factors (low socioeconomic status), and cultural factors (increased acculturation) are nonmodifiable factors. Behavioral factors related to diet (sugary beverages, increased energy intake) and psychological factors (increased stress) are modifiable [33]. Among modifiable risk factors, sugar consumption is considered one of the most important factors associated with both conditions [17]. Therefore, a high prevalence of dental caries and obesity and their common risk factor etiology call for the integration and collaboration of strategies aimed at preventing and controlling both conditions locally and globally.

There were certain limitations to the study. Data were collected from male public schools; hence, care should be exercised when generalizing the results to female students and private school children. Due to cultural norms in the country, male researchers could only access to male schools. Only the father's education was obtained in the study. However, physical activities, consumption of food, family history of systemic conditions (obesity linked with cardiovascular disease and diabetes), and socioeconomic influences were controlled when assessing the association between caries and obesity. However, the temporality of the association cannot be ascertained in a cross-sectional study design. Conflicting results in the literature about the direction and effect size of the association between increased weight and dental caries is due to heterogeneity in the methodologies of studies, sampling techniques, clinical measurement parameters, and statistical methods. Therefore, it is suggested that longitudinal studies should be conducted to better understand the possible relationship between caries and obesity in children.

## 5. Conclusion

The prevalence of caries and obesity was high in this male adolescent sample. The obese children demonstrated significantly higher untreated caries and caries experience than nonobese children. The children with high caries experience were 2.21 times more likely to have obesity than children with low caries experiences. No/school education of father, family history of obesity, and not performing physical activity were significantly associated with an increased likelihood of obesity. Preventive programs and policies should address public health issues related to caries and obesity in male teenagers.

## Figures and Tables

**Table 1:** Characteristics of study participants.

Study variables	*N* (%) (*N* = 258)
*Father's education*	
No/school education	172 (66.7)
College/university education	86 (33.3)

*Monthly family income*	
Low/medium income	145 (56.2)
High income	113 (43.8)

*Performed physical activity*	
Yes	230 (89.1)
No	28 (10.9)

Obese family member	102 (39.5)

Family history of systemic disease	101 (39.1)

Prevalence of caries	206 (79.8)

Age	Mean ± SD
	14.29 ± 1.11

DMFT	3.55 ± 2.94

BMI	23.42 ± 6.82

**Table 2:** Relationship between BMI and dental caries experience among study participants.

*Four categories based on BMI*	DMFT (mean/SD)	*P* value	Untreated caries	*P* value
Underweight (BMI < 18.5)	3.04 ± 2.77	0.106	2.71 ± 2.55	0.046^*∗*^
Normal (BMI 18.5–24.9)	3.61 ± 2.8		3.31 ± 2.71	
Overweight (BMI 25–30)	3.27 ± 2.7		2.82 ± 2.69	
Obese (BMI > 30)	4.38 ± 3.53		4.11 ± 3.22	

*Two categories based on BMI*	DMFT (mean/SD)	*P* value	Untreated caries	*P* value

Underweight, normal weight, and overweight (BMI < 18.5–30)	3.35 ± 2.77	0.021^*∗*^	3.01 ± 2.66	0.010^*∗*^
Obese (BMI > 30)	4.46 ± 3.54		4.17 ± 3.22	

**Table 3:** Association of caries experience and other factors with obesity in study participants.

Study variables	Unadjusted odds ratio (95% confidence interval)	*P* value	Unadjusted odds ratio (95% confidence interval)	*P* value
*Father's education*	3.31 (1.41, 7.75)	0.004	3.43 (1.29, 9.12)	0.014
No/school education^*∗*^				
College/university education				

*Monthly family income*	1.13 (0.59, 2.16)	0.707	0.84 (0.39, 1.81)	0.665
Low income^*∗*^				
High income				

*Caries experience (DMFT)*	2.34 (1.22, 4.51)	0.01	2.33 (1.07, 5.04)	0.032
Low caries experience (0–4)				
High caries experience (5–15)^*∗*^				

*Having obese family member*	2.6 (1.35, 4.98)	0.003	3.12 (1.47, 6.63)	0.003
Yes^*∗*^				
No				

*Family history of systemic disease*	1.54 (0.81, 2.93)	0.183	1.75 (0.82, 3.76)	0.149
Yes^*∗*^				
No				

*Perform physical activity*	4.32 (1.88, 9.94)	<0.001	4.95 (1.9, 12.89)	0.001
Yes				
No^*∗*^				

*Number of meals per day*	0.4 (0.16, 0.99)	0.042	0.20 (0.07, 0.60)	0.004
1–3 meals per day				
4 and more meals per day^*∗*^				

*Soft drinks per week*	1.21 (0.59, 2.47)	0.597	1.11 (0.42, 2.89)	0.839
0–3 times per week				
4 and more per week^*∗*^				

*Fast food per week*	1.32 (0.58, 3.03)	0.505	2.43 (0.79, 7.45)	0.119
0–3 times per week				
4 and more per week^*∗*^				

^*∗*^Reference category.

**Table 4:** Multiple logistic regression final model (backward stepwise (likelihood ratio)): association of caries experience and other factors with obesity in study participants.

Study variables	Adjusted odds ratio (OR)	*P* value
*Father's education*	3.54 (1.34, 9.37)	0.011
No/school education^*∗*^		
College/university education		

*Caries experience (DMFT)*	2.21 (1.04, 4.71)	0.04
Low caries experience (0–4)		
High caries experience (5–15)^*∗*^		

*Having an obese family member*	3.27 (1.55, 6.89)	0.002
Yes^*∗*^		
No		

*Perform physical activity*	4.37 (1.73, 11.07)	0.002
Yes		
No^*∗*^		

*Number of meals per day*	0.217 (0.08, 0.62)	0.004
1–3 meals per day		
4 and more meals per day^*∗*^		

*Fast food per week*	2.65 (0.97, 7.26)	0.058
0–3 times per week		
4 and more per week^*∗*^		

^*∗*^Reference category.

## Data Availability

The SPSS data file of this study is available from the corresponding author upon request.
